# “Who is watching the watchdog?”: ethical perspectives of sharing health-related data for precision medicine in Singapore

**DOI:** 10.1186/s12910-020-00561-8

**Published:** 2020-11-19

**Authors:** Tamra Lysaght, Angela Ballantyne, Vicki Xafis, Serene Ong, Gerald Owen Schaefer, Jeffrey Min Than Ling, Ainsley J. Newson, Ing Wei Khor, E. Shyong Tai

**Affiliations:** 1 Centre for Biomedical Ethics, Yong Loo Lin School of Medicine, National University of Singapore, Singapore, Singapore; 2 Department of Primary Health Care and General Practice, University of Otago, Dunedin, New Zealand; 3 Faculty of Science, National University of Singapore, Singapore, Singapore; 4 Sydney Health Ethics, Faculty of Medicine and Health, Sydney School of Public Health, University of Sydney, Camperdown, Australia; 5 Department of Medicine,Yong Loo Lin School of Medicine, National University of Singapore, Singapore, Singapore; 6 Saw Swee Hock School of Public Health, National University of Singapore, Singapore, Singapore; 7 Duke-National University of Singapore Graduate Medical School, Singapore, Singapore

**Keywords:** Data sharing, Bioethics, Governance, Precision medicine, Public attitudes, Public trust, Singapore, Qualitative research

## Abstract

**Background:**

We aimed to examine the ethical concerns Singaporeans have about sharing health-data for precision medicine (PM) and identify suggestions for governance strategies. Just as Asian genomes are under-represented in PM, the views of Asian populations about the risks and benefits of data sharing are under-represented in prior attitudinal research.

**Methods:**

We conducted seven focus groups with 62 participants in Singapore from May to July 2019. They were conducted in three languages (English, Mandarin and Malay) and analysed with qualitative content and thematic analysis.

**Results:**

Four key themes emerged: nuanced understandings of data security and data sensitivity; trade-offs between data protection and research benefits; trust (and distrust) in the public and private sectors; and governance and control options. Participants were aware of the inherent risks associated with data sharing for research. Participants expressed conditional support for data sharing, including genomic sequence data and information contained within electronic medical records. This support included sharing data with researchers from universities and healthcare institutions, both in Singapore and overseas. Support was conditional on the perceived social value of the research and appropriate de-identification and data security processes. Participants suggested that a data sharing oversight body would help strengthen public trust and comfort in data research for PM in Singapore.

**Conclusion:**

Maintenance of public trust in data security systems and governance regimes can enhance participation in PM and data sharing for research. Contrary to themes in much prior research, participants demonstrated a sophisticated understanding of the inherent risks of data sharing, analysed trade-offs between risks and potential benefits of PM, and often adopted an international perspective.

Precision medicine (PM) aims to provide more personalised care to patients, taking account of genomic, environmental and behavioral information [1]. In general, medical knowledge has been largely built on physiological, epidemiological and clinical trial data, reflecting the health status and treatment response of entire communities. The overarching aim of PM is to use genomic analyses and data analytics to build a finer grained and tailored approach to predicting disease progression and treatment response for individual patients [2, 3]. For this paradigm to work, researchers would require access to comprehensive clinical data and biological material from many people to be able to identify patterns and stratify small groups of patients. Key examples of PM initiatives include the United States’ *All of Us programme* which seeks to recruit one million volunteers to share genomic samples and data for research [4]; and the China Precision Medicine Initiative, with an estimated investment of US$9.2 billion over the next 15 years (2016–2030) [5].

PM initiatives typically seek broad consent when participants enrol and do not seek specific consent for every time the data or samples are shared with a new research team. Health data sharing for research under this consent model should operate within the social licence and in line with public expectations. The concept of a social licence refers to a privilege to operate, implicitly granted by society to an organisation or profession, often in the absence of explicit consent [6]. It refers to a practice that broader publics are willing to accept as morally and socially permissible. Identifying the boundaries of the social licence is difficult; and this is often most evident when activities overstep this boundary and there is a subsequent public backlash [7]. A breach of public trust and expectations can have wide-ranging negative consequences for a PM initiative. Such concerns materialised in the wide-spread rejection of the proposed UK Care.data project 2014 [7, 8]. It is thus important to characterise the boundaries and nature of the social licence for health data sharing for research in advance.

In this study, we examine public expectations and concerns about health data sharing for PM in Singapore. This small city-state of 5.7 million inhabitants has a high gross domestic product, a robust and comprehensive health system, and strong investment in biomedical research [9, 10]. Singapore has invested significantly in developing PM with the large-scale, whole-genome sequencing of Singapore’s multi-ethnic Asian population (SG10k) [11]. Some of this data has been linked to patients’ electronic medical records (EMR) and used to support clinical care under the SingHealth Duke-NUS Institute of PRecISion Medicine (PRISM) initiative [12]. Singapore has the capacity to upscale this initiative across the public healthcare system, which is served by a national IT provider that can integrate health information from multiple sources, including EMR, biobanks and administrative databases [13]. This combination of comprehensive health information, and expertise in genomics research and clinical implementation [14], puts Singapore in a strong position to initiate a nation-wide PM programme and contribute to similar initiatives internationally.

Despite these strengths, however, several major high-profile data breaches in Singapore have the potential to undermine efforts to establish an effective PM programme. These included unauthorised access to 1.6 million patient records held at SingHealth (one of the three major health clusters) and a leak involving the names of over 14,000 individuals in the HIV positive registry [15, 16]. These leaks involved identifiable clinical data; even though they were not directly related to precision medicine, or even research, they may have increased public anxiety around health data sharing. Maintaining the social licence for PM thus requires responding to community concerns about data security and ethical issues. This study aimed to examine those concerns and seek views on potential governance strategies for managing them.

## Background

There is a growing body of international empirical literature exploring the views of patients and various publics about sharing clinical data and biological samples for health research. The emerging consensus shows broad conditional support for sharing health data for research. Four common themes are reported in this literature: (1) the necessity of appropriate governance structures; (2) that research aims to produce public benefit; (3) the reluctance to allow commercialisation of public data sets, and (4) concerns about privacy and data security. A recent meta-analysis of this literature found the following recurrent issues: social value of the research, privacy, risk-minimisation, data security, control, transparency and engagement, trust, as well as responsibility and accountability [17].

Prior empirical research in Singapore indicates patient preferences for broad consent models to manage genomic data sharing for research and the return of cancer profiling results (provided this is accompanied with adequate explanation) [18]. Broad consent involves consent for an unspecified range of future research projects, subject to limited content and/or process restrictions, collected at the point of sample or data donation; it is popular for bio-banking globally [19]. It contrasts with specific consent models where consent is sought from donors each time data are accessed for specific research projects. While specific consent provides data donors with greater individual-level control over who gets access to the data, broad consent models are more common because they reduce the costs of research and burdens on donors being repeatedly re-contacted for consent [20].

There are three key limitations of the current empirical literature about attitudes to data sharing. First, the findings quickly become outdated because the technology and data ecosystem are changing so rapidly. As technology develops and the public gains more experience with the benefits and risks associated with data sharing, public understanding, expectations and concerns about data sharing are re-calibrated. For example, models of data de-identification in research continue to evolve. As more personal data becomes available in the public sphere there is greater likelihood of de-identified data being re-identified so de-identification methods and data regulation have to adjust. De-identification is a process of detecting and removing identifiers (e.g., personal names and national identification numbers) that directly or indirectly point to a person. True anonymization of datasets is becoming technically unachievable; and in several respects is undesirable for PM which ultimately seeks to integrate genomic research and clinical care [21, 22]. Therefore effective de-identification is an essential tool for protecting the privacy of participants in PM research [23].

Second, much of the existing literature investigates patient and public *preferences* about data sharing and patient control of data access and use [24–29]. This focus on preferences is arguably misdirected. Taylor and Taylor found that people under some circumstances were still willing to accept “non-preferred” data sharing models [30]. Therefore, rather than asking about preferences, it may be more relevant to ask: what do people expect from participating in data-driven medical research and what costs are they willing to bear to gain the purported benefits from data-driven medical research? This question is relevant because there is an inherent trade-off between competing values in managing access to data for research: most obviously between protecting privacy and generating public health benefits from data-driven research [31]. Research that simply asks participants what they want or prefer may generate an idealised but impractical model that does not sufficiently account for these trade-offs. Few prior studies explicitly explore which data sharing practices people are *willing* to accept, all things considered, as opposed to what they prefer [32–34].

Furthermore, the views of Asian populations around these issues are under-represented in the literature. Just as European genomes are over-represented in genomics [35], the majority of qualitative and quantitative studies about public attitudes and perspectives of genomics have been conducted in Europe, North America and Australia. Low rates of Asian minority participation in biobanks have been attributed to cultural views that blood is sacred [36] and fear of discrimination [37]. According to the Global Alliance for Genomics and Health, governance frameworks for the responsible sharing of genomic and health-related data should be culturally appropriate and sensitive to local norms, practices and perspectives [38]. It cannot be assumed that the perspectives people have about data sharing in Asian countries are the same as those reported elsewhere [39]. Therefore, we aimed to examine those perspectives in this qualitative study of the ethical concerns that Singaporeans have about sharing health-related data and what governance strategies could be put in place to address them.

## Methods

A commercial market research firm (Beacon Consulting Pte Ltd) was contracted to recruit participants, conduct the focus groups, and transcribe the discussions for analysis. The firm recruited participants with consecutive sampling from several online platforms (e.g. Facebook & Gumtree) using advertisements and an anonymous survey to screen prospective participants for their eligibility, availability and demographic profile (i.e. age, sex, education, ethnicity). Only Singaporean citizens or permanent residents over the age of 21 years were eligible to participate. This restriction was to reflect the recruitment parameters for precision medicine initiatives such as SG10K [11] in Singapore. Participants gave their written informed consent and received a supermarket voucher of SGD 50.00 for participation.

The study team developed the discussion guide in consultation with the research firm. The guide covered three topics: (1) concerns about the storage and sharing of data for PM; (2) potential governance strategies to mitigate or alleviate concerns; and (3) the kinds of benefits participants would expect from PM. At the commencement of each focus group (FG), participants were shown videos explaining the concepts of genes and precision medicine, and describing that data would be stored and shared only in a de-identified format but would be encoded to allow re-identification of an individual in the future, if needed (e.g. to withdraw them from a database or contact them with health-relevant findings). It also discussed the potential security risks from an unauthorised data breach and showed two cases that describe the potential uses and benefits of PM for treating individual patients. The videos shown to participants are available online [40].

Focus groups were conducted at the premises of the market research firm in three languages (English, Mandarin, and Malay) and were observed by at least one member of the project team. The duration of each focus group was between 90–130 mins. The facilitator avoided using the term *anonymous* and emphasised that data for PM would be stored and shared in a de-identified format. The firm transcribed the discussion from audio recordings and translated the non-English discussions into English (checked with reverse translation). They also removed any personal identifiers from the transcripts before sharing them with the study team for analysis. Transcripts were analysed in NVivo 12 (QSR International) using qualitative thematic analysis [41]. The coding was primarily shared between two senior researchers (TL and VX) with assistance from two graduate students (SO and JL) until thematic saturation was reached (where no new themes relevant to the research aims were emerging). Content was coded inductively for topics, which were progressively collapsed into broader thematic categories, and refined over several meetings between the coders and study team. Refer to Additional file 1 for COREX table with more information on methods.

## Results

**Fig. 1:**
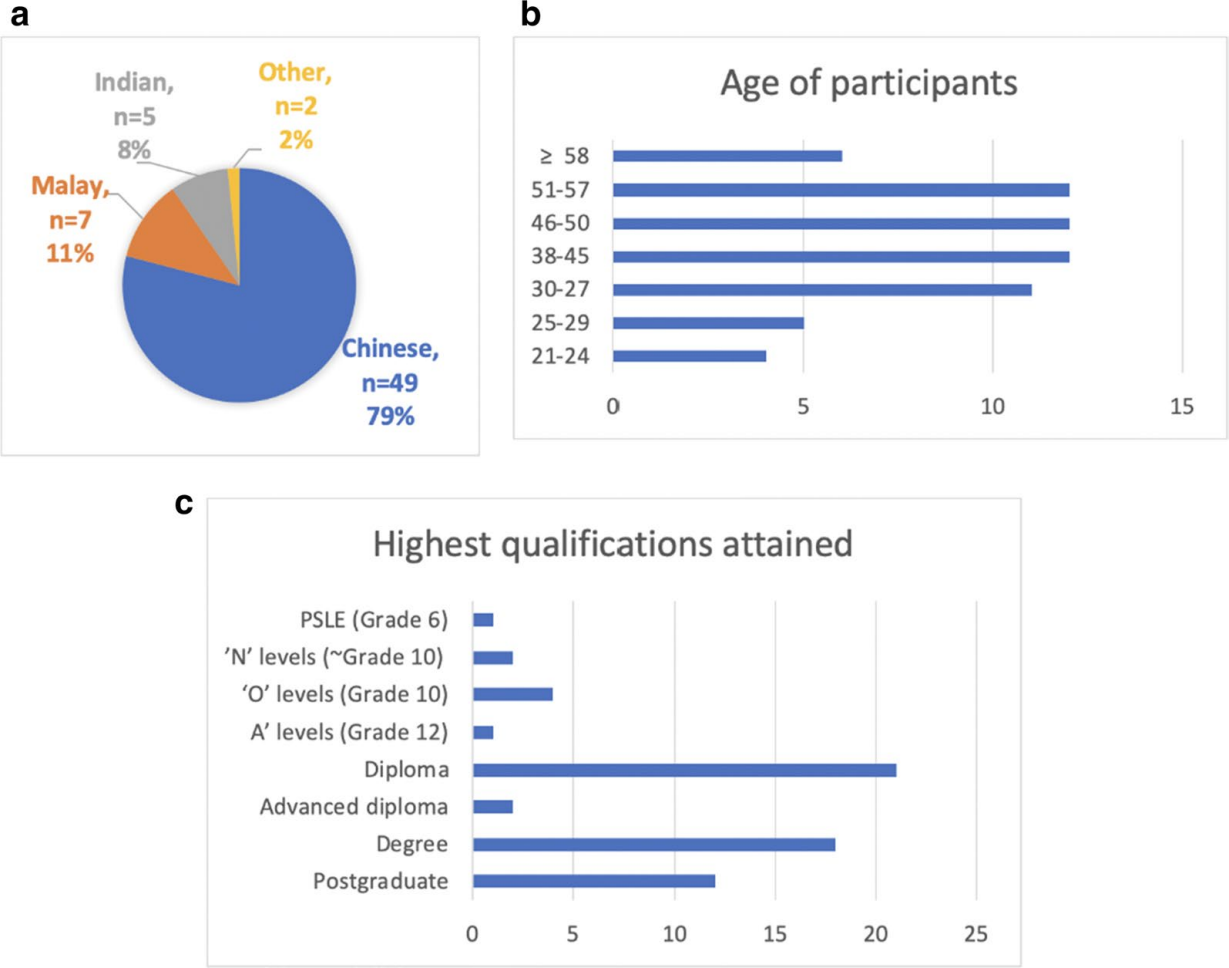
Demographic composition of the focus groups by ethnicity (**a**), age (**b**) and education (**c**)

Seven focus groups were conducted from May to July 2019: five in English, one in Mandarin, and another in Malay. Each focus group lasted between 90 and 120 min and had six to eleven people, totalling 62 participants for the study (see Fig. 1 for demographic composition). The median age range was 38 to 45 years. The majority were ethnic Chinese (49), with Malay (7) being the next largest group, and Indian (5) and others (2) making up the rest. The majority of participants were female (63%) and most had either a diploma (37.1%) or university degree (48.4%).

Despite efforts made to make clear the distinction between personal and de-identified data, and to avoid the term *anonymous*, participants regularly reverted to discussing personally identifiable data and anonymity. For example, immediately following a discussion where participants explicitly acknowledged that data would be de-identified, another expressed their concern that “It can be misused. It's my personal information so I would like to guard it” (FG0521_P1). Although initially out of scope, some comments relating to the sharing of personal information are included in the results as they reflect the participants’ concerns about data sharing, and it was impossible to completely disentangle conversations about personal and de-identified data.

The following results are organised around four key themes: nuanced understandings of data security and data sensitivity; trades-offs between data protection and research benefits; trust (and distrust) in the public and private sectors; and governance and control options.

### Nuanced understandings of data security and sensitivity

While participants were generally comfortable with the idea of storing and sharing de-identified data, concerns were raised about data security. The primary concern was about the potential misuse of data (e.g. for malevolent purposes, such as bioterrorism, discrimination or profiteering) and the resulting harm that might arise from a data breach. However, some participants appeared to have a good understanding of the various security measures that could be put in place to protect the identity of volunteers in a PM program and to reduce the likelihood of an unauthorised breach. Some drew on their experiences and knowledge of previous data breaches, with references to SingHealth and the HIV registry cases arising frequently and without prompting. These events gave participants a reference point for exploring the issues of data sensitivity and security. They also recognised that no data security systems were impervious to attacks and no data holders, including trusted government agencies, could guarantee data security.

Participants were generally more worried about the security of what they perceived to be sensitive data. People suffering stigmatised conditions in Singapore, such as infectious diseases like HIV or mental illness, were viewed as being more vulnerable to an unauthorised disclosure than someone who was in good health or was suffering from conditions such as cancer.

If I have like [a] generic health condition, perhaps like high cholesterol or heart issues, I wouldn't be so worried about my information being shared. But if […] I have HIV or things like that, that are more sensitive, then I would be concerned. But if its generic health situations, I, for me, personally, I won't be that concerned. (FG0530_P4).

However, opinions varied on the sensitivity of genetic health information. Some participants were relatively unconcerned about sharing genetic data. Others were worried about insurers, employers or school selectors using (personally identifiable) genetic data against the interests of the data donor:

[If you] happen to have some genetic diseases that could result in you having a higher risk of a certain disease and what happens if this information is sold to the insurance companies and then you will be anti-selection, you won't be covered for a certain disease or when your coverage will be very high. Then that would be a concern. (FG0530_P7).

Participants also recognised that some people might be vulnerable to unauthorised data breaches due to their environment or circumstances, rather than the sensitivity of the data itself. Public figures (e.g. celebrities and politicians) were viewed as greater targets for ‘hackers’ than ordinary citizens.

### Trades-offs between data protection and research benefits

There was broad support for sharing data with researchers at academic and healthcare institutions (both local and international) as they were perceived as conducting socially valuable research. Participants recognised and articulated the implicit trade-offs between protecting data from a breach, on the one hand, and promoting access to the data to generate potential benefits on the other hand.

We have to think about the benefits that you can reap from this programme, having the genomic data and sharing it through the national body as well as at an international level. That is basically for the goodness of humankind in the world. So the issue of transfer of information, stolen data, hackers, either for political use or used by pharmaceutical companies. They're bad, unfortunately […] that is a general concern. But I feel that the benefits, weighing the pros and cons, it is better to have this data. (FG0521_P2).

The purpose of data sharing was an important factor in the discussions. The introductory video provided examples of possible health benefits to individuals and their families. However, participants also went on to highlight a wider range of societal benefits for what was frequently termed, unprompted, the “greater good” (e.g. FG0527_P3&P6, FG0524_P2&P3, FG0530_P6&P2). These benefits included the development of new medicines, improved or more affordable healthcare, and improved understanding of genetic diseases (including hereditable cancer). Participants recognised the genomic research in Singapore could be especially valuable to minority ethnic groups in Singapore and Asia who may not be well represented in other PM initiatives.

Participants were concerned about parties with *authorised* access using the data for purposes contrary to the intentions or expectations of the data donors. Hence, it was important for them to understand the reasons why data would be shared with anyone inside or outside of the institution holding the data:

I guess, for me I think it's not so much the party accessing the data, but rather how is the data being used for and for what purpose. So knowing that, then I'm able to make a better decision as in whether I want to participate. …if it's from a big pharmacy company, then I think it may be for a commercial gain, but again it still help people. So I guess it's still the purpose, how the data being used, the purpose what is it used for. (FG0524_P11).

As indicated in the quote above, many participants expected that they would be notified prior and/or asked for consent for data-sharing. Some mentioned Singapore’s personal data protection laws (i.e. the PDPA) and expressed in strong terms that their consent should be sought before data were shared for any purpose. However, many others were willing to trust the institution storing the data to only allow access to trustworthy third parties for worthwhile purposes of generating benefit, and would not need to provide new consent to each research project.

So, if I [..] give you all my data, ok, so actually I trust you enough. (FG0530_P10).

### Trust (and distrust) in the public and private sectors

When asked about who they would trust to hold the data, participants suggested academic research institutions, and healthcare providers. Government agencies came through as trusted entities for ensuring data security. Given the health-relatedness of the data being stored, the Ministry of Health (MOH) specifically was frequently mentioned. This was despite some recognition that MOH had jurisdictional responsibility for data accessed in previous breaches. In addition to MOH, government defence and security agencies were also mentioned, along with statutory boards and public–private partnerships that regulate digital and information technologies in Singapore (e.g. iHIS [42] and GovTech [43]). Trust was not absolute, but rather participants expressed a nuanced approach, differentiating between different government agencies and different functions (e.g. data security, use and oversight).

On the other hand, there was significant debate amongst participants about sharing data with researchers at private or commercial entities, such as pharmaceutical companies. Some participants highlighted the fact that the pharmaceutical industry contributes to social value by developing new medicines; others were more ambivalent about the high costs of some medications in Singapore. Furthermore, participants were uncertain as to how the government could ensure that individuals or the public healthcare system would benefit from data-sharing with industry.

Commercial companies, ultimately they […] make money, so […] they [may] take your information, develop a certain diagnostic kit and then sell it off to other companies without benefitting the whole of Singaporeans and we are actually providing them with the data to come up with the kit, so I think […] they must bring some of the benefits back to the Singapore community. (FG0530_P7).

Overall, there was strong reluctance to share health data with private insurance companies. There was much discussion about the potential for insurance companies to use data to deny coverage to certain ethnic groups and/or increase the cost of insurance plans. By comparison, some participants supported sharing health data with insurance companies, arguing that they could use the data to benefit patients by tailoring plans for specific diseases or expanding coverage for patients not currently covered. Others did not see the need to share the data with insurance companies at all, noting that they could instead work with public research institutions as trustworthy actors to develop equitable insurance plans based on aggregated data.

### Governance and control options

Participants were asked to suggest strategies to ensure responsible and trustworthy approaches to storing and sharing health data. Participants made numerous suggestions for data security measures that would help to alleviate their concerns, such as block chain technology, data masking, two-step verification, and tiered access restrictions. The need for a strong regulatory environment was acknowledged. Some participants also felt that data should not be accessed without the specific consent of volunteers, although it is unclear if they were talking about personally identifiable data or de-identified data. Being able to withdraw from the PM initiative appeared to be important for many participants; although exactly what that meant (e.g. severing all links to data, deleting all records from the database, declining participation in a particular study) was not explored.

In addition, some participants suggested that independent oversight of data sharing in Singapore across sectors and agencies would be an important component of a trustworthy governance system. Several options were proposed to fulfil this oversight function, including non-government organisations or an intergovernmental agency within Singapore. Some suggested cross-sectorial “semi-government, private sector” (FG0528_P5) collaborations. International oversight bodies were also suggested in the context of government agencies gaining access to data:

Who is watching the watchdog? […] there has to be checks and balances from all different agencies… Or even, not even the agency, I think even bigger body. A bigger international body. … Like a United Nations or some nations right? Or Interpol, for example, I do not know. Beyond just our country. (FG0527_P8).

Thus, although there was a general sense that government agencies could be trusted to regulate the research institutions and private entities that access and use data within Singapore, there was also some unease with how the government itself might use the data in the absence of external oversight or checks and balances. The reasons for the apparent distrust were not probed and preferences for particular oversight mechanisms were not explored in any further depth.

## Discussion

Key findings show that participants in this study were generally comfortable with the idea of storing and sharing de-identified genomic and medical data (e.g. from EMRs) with researchers from universities and healthcare institutions, both in Singapore and overseas. However, this broad support was conditional on the likelihood of producing a tangible health benefit for patients and the adoption of acceptable de-identification and data security processes. Participants also raised a range of concerns about data sharing, the conditions under which they would accept it taking place and the trade-offs they made between risks and potential benefits.

Participants in this study were mostly tertiary educated. Thus, it is unsurprising that they demonstrated nuanced and sophisticated understandings of the ethical issues surrounding data security and sharing for PM. Participants took a pragmatic approach to data sharing and understood degrees of data sensitivity and limitations of security measures. They also appreciated that this was related to, but different from, data identifiability. That said, there remained persistent slippage between discussion of personal identifiable data and de-identified data, *even amongst this highly educated group* of people. The difficulty in holding personal data separate from de-identified data may have been because the distinction was not clear in the mind of participants and the short videos shown to participants may have been insufficient to facilitate deeper understandings. However, it also reflects ongoing academic, regulatory and technical debate about the conceptual boundaries of personal and non-personal data, as well as methods of identification, de-identification and re-identification, and the persistent confusion between these terms [44–46].

Participants did not expect a guarantee of anonymity or data security and they were willing to accept some risks in order to gain the potential benefits of PM. Participants were most motived to accept these inherent risks for research aimed at producing public benefit. Finally, participants offered rich and diverse suggestions to alleviate concerns about data sharing. Participants preferred a multi-tiered approach including control options for individuals (e.g. the ability to withdraw from the PM initiative), regulatory limits on users and uses of the data, as well as independent oversight. Numerous suggestions were made about the types of organisations that could be trusted in an oversight role with the government being cited most prominently.

The results of our research are novel in four respects. First, compared to prior research [46] our results demonstrated a high level of awareness of the inherent risks associated with data collection, the impossibility of ensuring data security to avert **all** risks, and challenges of de-identifying data. Previous research has demonstrated broad support for data sharing for health research [18] so long as data are de-identified [47, 48] and privacy is protected [49]; both of which are increasingly difficult conditions to guarantee. Participants in this study also expressed concerns about data security and identifiability, but demonstrated a nuanced and sophisticated understanding of data security. Participants recognised that all security systems were vulnerable to an attack and differentiated between a data breach that amounted to a technical privacy invasion versus a data breach resulting in harm. Importantly, support for data sharing was not conditional on a guarantee of data security.

Second, participants weighed data protection and individual patient control on the one hand (personal risk) and the benefits of sharing the data to promote health research on the other (public good). Only a small proportion of the existing empirical research captures the public’s efforts to evaluate these trade-offs [46, 50]. Participants in our study engaged in these complicated trade-offs unprompted. Understanding how people make these assessments is important because they may be concerned about data security; but may be nonetheless willing to accept the inherent risks of data collection, storage and sharing if this facilitates medical progress. In our study, participants framed the debate as a question of trade-offs and opportunity costs without being prompted. This balancing of harms and benefits revealed some sophistication in participants’ understanding of the ethical issues and the trade-offs that would be necessary to generate value from PM. Our results suggest that public understanding of biobanking and data sharing in technologically developed countries such as Singapore may be deepening and maturing.

Third, participants were relatively unconcerned with the prospect of international data sharing. Previous research has shown general public reticence to share data across national borders, and especially with respect to sharing genomic data [51] or biological samples [52] and/or data relating to Indigenous communities [53]. Participants in this study, however, recognised the benefits of international data sharing to drive research and innovation, and highlighted the value of generating knowledge that would benefit all humankind. Some even suggested international oversight as a potential governance option for PM, suggesting an international rather than exclusively domestic approach to PM. This attitude may reflect the role of Singapore as a global hub for international commerce, business and travel.

Finally, participants expressed relatively high levels of trust in government authorities to ensure data security. This perspective contrasts with other countries where confidence in governments to safeguard data security and oversee the responsible use of health-related information is less striking. Governmental and legal responses to the prior data breaches at SingHealth and the HIV registry, which included substantial investigations and rapid adoption of reform measures [54], may have influenced this attitude. However, it also speaks to cultural norms and the socio-political relationship that Singaporeans have with the government and ministerial agencies. Singaporeans have experienced soft authoritarian governance for over 55 years where a single party has been in power since independence; providing political stability, economic prosperity, security, and access to housing, healthcare and education, in exchange for restricted liberties and personal freedoms [55]. For the most part, the citizenry has accepted this situation and trusts the pragmatic paternalism that is characteristic of Singapore. This degree of trust would, in all likelihood, pass onto a national PM initiative.

### Building trustworthy governance

Securing the social license for PM initiatives to operate will require trustworthy systems of governance. As participants in one discussion asked, “who watches the watchdog?”. This question is a critical reflection upon the capacity of any single agency to protect and promote the common good [56]. As our results and the prior literature suggests [7], the social license for PM programmes is likely to be contingent upon assumptions that participation is voluntary and use of the data is governed by broadly accepted social and cultural values, such as reciprocity and public benefit. Independent agencies and oversight mechanisms play an important role in holding others accountable when breaches occur; and, therefore, are a significant component of a trustworthy system of governance. Examples of possible oversight roles include the National Data Guardian for Health and Social Care in England [57], the Privacy Commissioner in New Zealand [58], and the Australian Government’s Data Governance Framework 2020 [59].

Other solutions may be found in models of systemic governance. Recognising the ethical deficiencies that informed consent provides as a mechanism in preserving autonomy and protecting privacy in data-intensive research, systemic governance refers to an oversight pipeline that accounts for the multiplicity of actors and stakeholders involved in an entire health data eco-system [60]. Vayena and Blasimme propose a framework with six norms for systemic oversight: adaptivity, flexibility, monitoring, responsiveness, reflexivity, and inclusiveness. This framework fits within an adaptive governance approach that allows policymakers to respond to changing technological and social conditions, and align regulation with public expectations by including multiple actors in the design and monitoring of the oversight pipeline [61]. It encourages collaboration between regulators and the regulated as well as social learning across different levels and hierarchies of governance structures.

A critical aspect of any governance system is attention to ‘who’ holds decision making power within the system. This issue is particularly relevant to PM programmes because they are long-term propositions where the available technologies will change over time, along with potential usages and users; often in highly unpredictable ways. The norms and values implicit to the social license are also liable to change, or at least be re-interpreted and re-prioritised over time, and in ways that may differ from the original configuration when a programme is established. Furthermore, social injustices that expose segments of the population to vulnerabilities from data breaches and misuse can aggregate and become exacerbated in unforeseen ways. Governance approaches must be adaptive rather than static. It matters who has a role in interpreting and applying governance frameworks, in defining the public interest, and appropriate uses and users of PM data [62].

Ostrom has argued that nested governance allows oversight systems to be scaled appropriately in reaction to changing circumstances [63]. Multiple levels of governance captures the benefits of centralisation whilst still being responsive to specific social-ecological challenges. One example is the need for data governance systems to respond to growing demands for Indigenous data sovereignty [64]. For example, New Zealand has a centralised research database called the Integrated Data Infrastructure (IDI) holding comprehensive microdata about people, households and businesses and governed by Statistics New Zealand (Stats NZ) [65]. In response to local concerns about control over the use of Māori data, Stats NZ has adopted an additional layer of review for research using Māori data, called Ngā Tikanga Paihere [66]. This review process was developed by leading Māori scholars and is an example of power sharing within a data governance model.

## Limitations and future research

Caution should be taken in extrapolating findings from these focus groups to the wider population in Singapore and beyond. As with any qualitative study, the results are not intended to be generalizable and may not reflect the views of all Singaporeans. Recruiting from social media platforms may only reach a segment of the population with access to the technology, and our study only included citizens and permanent residents. Approximately 30% of the population in Singapore are foreigners, with many being unskilled transient workers whose perspectives and understandings may differ greatly from those included in our study. Almost half participants in our study were tertiary educated with university degrees, compared with 32.4% university educated and 25% with primary education in the resident population [67]. Higher levels of education within our research population may explain their familiarity with data security, previous data leaks, and ability to engage in sophisticated trading-off between the benefits and risks of data sharing for PM. The lack of conceptual clarity around identification and de/re-identification also suggests that care should be taken in drawing conclusions about what data Singaporeans will be comfortable sharing, especially under a broad consent model.

Yet these findings provide important insights into ethical concerns that are held amongst Singaporeans and the trade-offs they may be willing to accept for data sharing. They have also identified several measures that could be applied in building a culturally appropriate trustworthy governance framework for PM in Singapore. Further research is needed to elaborate on the measures that Singaporeans would consider most important in the configuration of governing norms and values, and to spell out more explicitly where the moral boundaries of the social license will lie.

## Conclusion

Maintenance of public trust in data security systems and governance regimes can enhance participation in PM and data sharing for research. Our research sought to explore the boundaries of the social licence for data sharing for PM in Singapore. We were interested specifically in what data sharing arrangements participants would be willing to accept; as opposed to models they might prefer. Despite recent data security breaches, government agencies remain preferred guardians for health data but there was recognition that they too must be subject to oversight. The purpose of data sharing emerged as a primary factor affecting willingness to share data. Participants were comfortable with data sharing with academic and healthcare institutions (in Singapore or overseas) because they trust such researchers to produce socially valuable knowledge, products and interventions. There was general hesitancy to share data with industry and insurance companies but also recognition of the important role pharmaceutical companies and insurers play in drug development and healthcare. Participants suggested that a data sharing oversight body would help strengthen public trust and comfort in data research for PM in Singapore.

## Supplementary information

**Additional file 1**. COREX table.

## Data Availability

The datasets generated and/or analysed during the current study are not publicly available for reasons of personal privacy, but are available from the corresponding author on reasonable request.

## Abbreviations

PM: Precision medicine
EMR: Electronic medical records
PRISM: SingHealth Duke-NUS Institute of PRecISion Medicine
