# Supplementary material for: “Who is watching the watchdog?”: ethical perspectives of sharing health-related data for precision medicine in Singapore
Source: BMC Med Ethics. 2020 Nov 19;21:118. doi: 10.1186/s12910-020-00561-8 (PMC7678103; doi:10.1186/s12910-020-00561-8)
Supplement: Supplementary file 1 — Additional file 1. COREX table. [file 12910_2020_561_MOESM1_ESM.docx]

Supplementary Table: COREQ checklist

| COREQ checklist |  | Location in manuscript (Section, page no.) |
| --- | --- | --- |
| **Domain 1: Research team and reflexivity** | | |
| **Personal Characteristics** | | |
| 1. Interviewer/facilitator  Which author/s conducted the interview or focus group? | The focus groups were recruited by a market research firm and facilitated by professional facilitators employed at the firm. Three different facilitators conducted the research: ZB (English language), SL (Mandarin), and MF (Malay). Professional facilitators were chosen over content experts to cater for multiple languages and the idiosyncratic syntaxes of the (Singaporean) participants. | Supp materials |
| 2. Credentials  What were the researcher’s credentials? E.g. PhD, MD | ZB: Bachelor’s degree  SL: Bachelor’s degree  MF: Diploma | Supp materials |
| 3. Occupation  What was their occupation at the time of the study? | ZB: research and training consultant  SL: head of research department  MF: senior training consultant | Supp materials |
| 4. Gender  Was the researcher male or female? | ZB: M  SL: F  MF: F | Supp materials |
| 5. Experience and training  What experience or training did the researcher have? | ZB: 25 years working experience, 23 years as a professional facilitator  SL: 10 years working experience, 8 years as a professional facilitator  MF: 19 years working experience, 4 years as a professional facilitator | Supp materials |
| **Relationship with Participants** | | |
| 6. Relationship established  Was a relationship established prior to study commencement? | No. | Supp materials |
| 7. Participant knowledge of the interviewer What did the participants know about the researcher? E.g. personal goals, reasons for doing the research | Participants were introduced to the facilitators at the commencement of the focus group, their position in the firm and role in facilitating the focus groups on behalf of the research team. | Supp materials |
| 8. Interviewer characteristics What characteristics were reported about the interviewer/facilitator? E.g. bias, assumptions, reasons and interests in the research topic | The professional facilitators had no interests in the research topic or other sources of bias. | Supp materials |
| **Domain 2: Study design** | | |
| **Theoretical framework** | | |
| 9. Methodological orientation and Theory  What methodological orientation was stated to underpin the study? e.g. grounded theory, discourse analysis, ethnography, phenomenology, content analysis | Qualitative thematic analysis | P7-8 |
| **Participant selection** | | |
| 10. Sampling How were participants selected? E.g. purposive, convenience, consecutive, snowball | Participants were recruited using consecutive sampling online (facebook ads, online recruitment platforms which are open to the public), and through the firm’s panel database. | P7 |
| 11. Method of approach How were participants approached? E.g. face-to-face, telephone, mail, email | Participants were first asked to fill in an online form. Recruiters then screened participants via phone so ensure that they meet inclusion criteria (> 21 years, citizen or PR, willingness to participate) and quota (educational background, preferred language). | Partly on P7 and Supp materials |
| 12. Sample size How many participants were in the study? | 62 participants. | P8 |
| 13. Non-participants How many people refused to participate or dropped out? Reasons? | Drop-outs: 16.  Reason given for dropping out: schedule conflicts. | Supp materials |
| **Setting** | | |
| 14. Setting of data collection Where was the data collected? E.g. home, clinic, workplace | Data was collected in a professional setting; a meeting room in the market firm’s office. | P7 |
| 15. Presence of non-participants  Was anyone else present besides the participants and researchers? | No. | Supp materials |
| 16. Description of sample  What are the important characteristics of the sample? e.g. demographic data, date | Demographic characteristics include gender, age range, highest educational qualification.  Data was collected between: 21 May 2019 and 28 June 2019. | P8 and P20 (Figure 1) |
| **Data collection** | | |
| 17. Interview guide Were questions, prompts, guides provided by the authors? Was it pilot tested? | Focus groups were semi-structured using an interview guides and prompts. The guide was not pilot tested. | Structure of the guide is on P7 |
| 18. Repeat interviews  Were repeat interviews carried out? If yes, how many? | No. | Supp materials |
| 19. Audio/visual recording  Did the research use audio or visual recording to collect the data? | The focus groups were audio recorded for transcription. The firm also used video recording to facilitate transcription, which was deleted once completed | P7 and Supp materials |
| 20. Field notes  Were field notes made during and/or after the interview or focus group? | An assistant (market research firm) made the field notes to aid transcription.  Members of research team (TL, OS, SO) took turns to observe focus groups and take notes. | Supp materials |
| 21. Duration What was the duration of the interviews or focus group? | The focus groups lasted between 1h 30 to 2h 10mins. | P7 |
| 22. Data saturation  Was data saturation discussed? | Yes. | P8 |
| 23. Transcripts returned Were transcripts returned to participants for comment and/or correction? | No. Participants were de-identified and consented to not being re-contacted again. | Supp materials |
| **Domain 3: analysis and findings** | | |
| **Data analysis** | | |
| 24. Number of data coders  How many data coders coded the data? | Four coders coded the data: TL, VX, SO, JL. | P8 |
| 25. Description of the coding tree  Did authors provide a description of the coding tree? | No. Can publish the coding tree upon request | Supp materials |
| 26. Derivation of themes  Were themes identified in advanced or derived from data? | Themes inductively emerged from the data. | P8 |
| 27. Software What software, if applicable, was used to manage the data? | QSR NVivo 12. | P8 |
| 28. Participant checking  Did participants provide feedback on the findings? | No. Participants were de-identified and consented to not being re-contacted again. | Supp materials |
| **Reporting** | | |
| 29. Quotations presented  Were participant quotations presented to illustrate the themes / findings? Was each  quotation identified? e.g. participant number | Yes, participant quotations were presented to support themes. Each quotation was identified by participant number and focus group. | Pages 9-12 |
| 30. Data and findings consistent  Was there consistency between the data presented and the findings? | Yes. We believe so. |  |
| 31. Clarity of major themes  Were major themes clearly presented in the findings? | Yes. We believe so. |  |
| 32. Clarity of minor themes Is there a description of diverse cases or discussion of minor themes? | Yes. We believe so. |  |
